# Supplementary material for: Pyruvate Dehydrogenase Kinase 1 inhibition mediated oxidative phosphorylation enhancement in cartilage promotes osteoarthritis progression
Source: BMC Musculoskelet Disord. 2023 Jul 20;24:597. doi: 10.1186/s12891-023-06585-6 (PMC10357736; doi:10.1186/s12891-023-06585-6)
Supplement: Supplementary file 1 — Additional file 1. [file 12891_2023_6585_MOESM1_ESM.zip › KEGG.Gsea.1653622432464/KEGG_SYSTEMIC_LUPUS_ERYTHEMATOSUS.html]

Details for gene set KEGG\_SYSTEMIC\_LUPUS\_ERYTHEMATOSUS[GSEA]

|  || Dataset | 55457\_collapsed\_to\_symbols.cls.cls #OA\_versus\_normal.cls.cls #OA\_versus\_normal\_repos |
| Phenotype | cls.cls#OA\_versus\_normal\_repos |
| Upregulated in class | OA |
| GeneSet | KEGG\_SYSTEMIC\_LUPUS\_ERYTHEMATOSUS |
| Enrichment Score (ES) | 0.23289938 |
| Normalized Enrichment Score (NES) | 1.0864015 |
| Nominal p-value | 0.27142859 |
| FDR q-value | 0.48710123 |
| FWER p-Value | 1.0 |
Table: GSEA Results Summary

  

Fig 1: Enrichment plot: KEGG\_SYSTEMIC\_LUPUS\_ERYTHEMATOSUS      
 Profile of the Running ES Score & Positions of GeneSet Members on the Rank Ordered List

  

| SYMBOL | TITLE | RANK IN GENE LIST | RANK METRIC SCORE | RUNNING ES | CORE ENRICHMENT || 1 | H2BC15 | H2B clustered histone 15 [Source:HGNC Symbol;Acc:HGNC:4749] | 407 | 0.704 | -0.0030 | Yes |
| 2 | C4A | complement C4A (Rodgers blood group) [Source:HGNC Symbol;Acc:HGNC:1323] | 632 | 0.611 | 0.0045 | Yes |
| 3 | FCGR1A | Fc fragment of IgG receptor Ia [Source:HGNC Symbol;Acc:HGNC:3613] | 633 | 0.610 | 0.0295 | Yes |
| 4 | HLA-DMB | "major histocompatibility complex, class II, DM beta [Source:HGNC Symbol;Acc:HGNC:4935]" | 679 | 0.598 | 0.0505 | Yes |
| 5 | CD86 | CD86 molecule [Source:HGNC Symbol;Acc:HGNC:1705] | 689 | 0.596 | 0.0742 | Yes |
| 6 | C8B | complement C8 beta chain [Source:HGNC Symbol;Acc:HGNC:1353] | 1001 | 0.517 | 0.0710 | Yes |
| 7 | SNRPD3 | small nuclear ribonucleoprotein D3 polypeptide [Source:HGNC Symbol;Acc:HGNC:11160] | 1046 | 0.508 | 0.0884 | Yes |
| 8 | GRIN2A | glutamate ionotropic receptor NMDA type subunit 2A [Source:HGNC Symbol;Acc:HGNC:4585] | 1054 | 0.506 | 0.1086 | Yes |
| 9 | HLA-DQA1 | "major histocompatibility complex, class II, DQ alpha 1 [Source:HGNC Symbol;Acc:HGNC:4942]" | 1334 | 0.452 | 0.1053 | Yes |
| 10 | FCGR2C | Fc fragment of IgG receptor IIc (gene/pseudogene) [Source:HGNC Symbol;Acc:HGNC:15626] | 1460 | 0.435 | 0.1133 | Yes |
| 11 | HLA-DMA | "major histocompatibility complex, class II, DM alpha [Source:HGNC Symbol;Acc:HGNC:4934]" | 1769 | 0.388 | 0.1051 | Yes |
| 12 | TRIM21 | tripartite motif containing 21 [Source:HGNC Symbol;Acc:HGNC:11312] | 1937 | 0.368 | 0.1072 | Yes |
| 13 | FCGR2B | Fc fragment of IgG receptor IIb [Source:HGNC Symbol;Acc:HGNC:3618] | 2016 | 0.360 | 0.1158 | Yes |
| 14 | H2AB3 | H2A.B variant histone 3 [Source:HGNC Symbol;Acc:HGNC:14455] | 2090 | 0.351 | 0.1244 | Yes |
| 15 | TNF | tumor necrosis factor [Source:HGNC Symbol;Acc:HGNC:11892] | 2098 | 0.350 | 0.1382 | Yes |
| 16 | HLA-DOB | "major histocompatibility complex, class II, DO beta [Source:HGNC Symbol;Acc:HGNC:4937]" | 2147 | 0.344 | 0.1486 | Yes |
| 17 | H4C1 | H4 clustered histone 1 [Source:HGNC Symbol;Acc:HGNC:4781] | 2270 | 0.330 | 0.1525 | Yes |
| 18 | H3C10 | H3 clustered histone 10 [Source:HGNC Symbol;Acc:HGNC:4775] | 2287 | 0.328 | 0.1647 | Yes |
| 19 | H2AZ2 | H2A.Z variant histone 2 [Source:HGNC Symbol;Acc:HGNC:20664] | 2341 | 0.322 | 0.1738 | Yes |
| 20 | CTSG | cathepsin G [Source:HGNC Symbol;Acc:HGNC:2532] | 2457 | 0.307 | 0.1774 | Yes |
| 21 | ACTN2 | actinin alpha 2 [Source:HGNC Symbol;Acc:HGNC:164] | 2532 | 0.299 | 0.1838 | Yes |
| 22 | H4C12 | H4 clustered histone 12 [Source:HGNC Symbol;Acc:HGNC:4784] | 2736 | 0.276 | 0.1793 | Yes |
| 23 | H3-4 | H3.4 histone [Source:HGNC Symbol;Acc:HGNC:4778] | 2751 | 0.275 | 0.1894 | Yes |
| 24 | H2AZ1 | H2A.Z variant histone 1 [Source:HGNC Symbol;Acc:HGNC:4741] | 2756 | 0.275 | 0.2004 | Yes |
| 25 | C3 | complement C3 [Source:HGNC Symbol;Acc:HGNC:1318] | 2764 | 0.273 | 0.2110 | Yes |
| 26 | HLA-DQB1 | "major histocompatibility complex, class II, DQ beta 1 [Source:HGNC Symbol;Acc:HGNC:4944]" | 2840 | 0.266 | 0.2161 | Yes |
| 27 | H2AC8 | H2A clustered histone 8 [Source:HGNC Symbol;Acc:HGNC:4724] | 2846 | 0.266 | 0.2265 | Yes |
| 28 | C1QA | complement C1q A chain [Source:HGNC Symbol;Acc:HGNC:1241] | 2902 | 0.260 | 0.2329 | Yes |
| 29 | H4C3 | H4 clustered histone 3 [Source:HGNC Symbol;Acc:HGNC:4787] | 3098 | 0.240 | 0.2275 | No |
| 30 | C1QB | complement C1q B chain [Source:HGNC Symbol;Acc:HGNC:1242] | 3365 | 0.215 | 0.2155 | No |
| 31 | H2BC6 | H2B clustered histone 6 [Source:HGNC Symbol;Acc:HGNC:4753] | 3377 | 0.214 | 0.2234 | No |
| 32 | H2BC3 | H2B clustered histone 3 [Source:HGNC Symbol;Acc:HGNC:4751] | 3708 | 0.185 | 0.2052 | No |
| 33 | MACROH2A2 | macroH2A.2 histone [Source:HGNC Symbol;Acc:HGNC:14453] | 3793 | 0.178 | 0.2059 | No |
| 34 | H4C8 | H4 clustered histone 8 [Source:HGNC Symbol;Acc:HGNC:4788] | 3811 | 0.175 | 0.2117 | No |
| 35 | H2BC5 | H2B clustered histone 5 [Source:HGNC Symbol;Acc:HGNC:4747] | 3895 | 0.165 | 0.2120 | No |
| 36 | C8A | complement C8 alpha chain [Source:HGNC Symbol;Acc:HGNC:1352] | 3918 | 0.163 | 0.2170 | No |
| 37 | H2BC7 | H2B clustered histone 7 [Source:HGNC Symbol;Acc:HGNC:4752] | 4026 | 0.153 | 0.2149 | No |
| 38 | C7 | complement C7 [Source:HGNC Symbol;Acc:HGNC:1346] | 4078 | 0.148 | 0.2170 | No |
| 39 | H2AW | H2A.W histone [Source:HGNC Symbol;Acc:HGNC:20507] | 4143 | 0.143 | 0.2178 | No |
| 40 | H2AJ | H2A.J histone [Source:HGNC Symbol;Acc:HGNC:14456] | 4204 | 0.137 | 0.2187 | No |
| 41 | HLA-DOA | "major histocompatibility complex, class II, DO alpha [Source:HGNC Symbol;Acc:HGNC:4936]" | 4344 | 0.125 | 0.2130 | No |
| 42 | C5 | complement C5 [Source:HGNC Symbol;Acc:HGNC:1331] | 4387 | 0.122 | 0.2147 | No |
| 43 | H4C14 | H4 clustered histone 14 [Source:HGNC Symbol;Acc:HGNC:4794] | 4450 | 0.117 | 0.2146 | No |
| 44 | H4C5 | H4 clustered histone 5 [Source:HGNC Symbol;Acc:HGNC:4790] | 4538 | 0.110 | 0.2124 | No |
| 45 | CD28 | CD28 molecule [Source:HGNC Symbol;Acc:HGNC:1653] | 4543 | 0.110 | 0.2165 | No |
| 46 | FCGR3A | Fc fragment of IgG receptor IIIa [Source:HGNC Symbol;Acc:HGNC:3619] | 4594 | 0.105 | 0.2169 | No |
| 47 | H2BC9 | H2B clustered histone 9 [Source:HGNC Symbol;Acc:HGNC:4755] | 4859 | 0.083 | 0.1997 | No |
| 48 | C9 | complement C9 [Source:HGNC Symbol;Acc:HGNC:1358] | 5415 | 0.039 | 0.1579 | No |
| 49 | H2BC17 | H2B clustered histone 17 [Source:HGNC Symbol;Acc:HGNC:4758] | 5464 | 0.036 | 0.1556 | No |
| 50 | HLA-DPB1 | "major histocompatibility complex, class II, DP beta 1 [Source:HGNC Symbol;Acc:HGNC:4940]" | 5486 | 0.033 | 0.1554 | No |
| 51 | CD40LG | CD40 ligand [Source:HGNC Symbol;Acc:HGNC:11935] | 5529 | 0.030 | 0.1533 | No |
| 52 | H2AC6 | H2A clustered histone 6 [Source:HGNC Symbol;Acc:HGNC:4733] | 5687 | 0.017 | 0.1417 | No |
| 53 | H3C7 | H3 clustered histone 7 [Source:HGNC Symbol;Acc:HGNC:4773] | 5716 | 0.014 | 0.1401 | No |
| 54 | MACROH2A1 | macroH2A.1 histone [Source:HGNC Symbol;Acc:HGNC:4740] | 5953 | -0.002 | 0.1218 | No |
| 55 | H2AC16 | H2A clustered histone 16 [Source:HGNC Symbol;Acc:HGNC:4730] | 6113 | -0.014 | 0.1099 | No |
| 56 | H2BC10 | H2B clustered histone 10 [Source:HGNC Symbol;Acc:HGNC:4756] | 6298 | -0.027 | 0.0966 | No |
| 57 | H3C3 | H3 clustered histone 3 [Source:HGNC Symbol;Acc:HGNC:4768] | 6308 | -0.028 | 0.0971 | No |
| 58 | FCGR2A | Fc fragment of IgG receptor IIa [Source:HGNC Symbol;Acc:HGNC:3616] | 6329 | -0.031 | 0.0968 | No |
| 59 | IFNG | interferon gamma [Source:HGNC Symbol;Acc:HGNC:5438] | 6410 | -0.036 | 0.0920 | No |
| 60 | HLA-DRA | "major histocompatibility complex, class II, DR alpha [Source:HGNC Symbol;Acc:HGNC:4947]" | 6550 | -0.046 | 0.0830 | No |
| 61 | H3C6 | H3 clustered histone 6 [Source:HGNC Symbol;Acc:HGNC:4769] | 6782 | -0.061 | 0.0674 | No |
| 62 | CD40 | CD40 molecule [Source:HGNC Symbol;Acc:HGNC:11919] | 6825 | -0.065 | 0.0668 | No |
| 63 | H4C13 | H4 clustered histone 13 [Source:HGNC Symbol;Acc:HGNC:4791] | 6852 | -0.067 | 0.0675 | No |
| 64 | ELANE | "elastase, neutrophil expressed [Source:HGNC Symbol;Acc:HGNC:3309]" | 6862 | -0.068 | 0.0696 | No |
| 65 | H4C4 | H4 clustered histone 4 [Source:HGNC Symbol;Acc:HGNC:4782] | 6888 | -0.071 | 0.0706 | No |
| 66 | CD80 | CD80 molecule [Source:HGNC Symbol;Acc:HGNC:1700] | 6918 | -0.074 | 0.0713 | No |
| 67 | HLA-DRB1 | "major histocompatibility complex, class II, DR beta 1 [Source:HGNC Symbol;Acc:HGNC:4948]" | 7087 | -0.086 | 0.0617 | No |
| 68 | H2AC4 | H2A clustered histone 4 [Source:HGNC Symbol;Acc:HGNC:4734] | 7191 | -0.096 | 0.0576 | No |
| 69 | C6 | complement C6 [Source:HGNC Symbol;Acc:HGNC:1339] | 7316 | -0.103 | 0.0521 | No |
| 70 | H3-3B | H3.3 histone B [Source:HGNC Symbol;Acc:HGNC:4765] | 7351 | -0.106 | 0.0538 | No |
| 71 | H3C12 | H3 clustered histone 12 [Source:HGNC Symbol;Acc:HGNC:4774] | 7371 | -0.108 | 0.0568 | No |
| 72 | H2BC21 | H2B clustered histone 21 [Source:HGNC Symbol;Acc:HGNC:4760] | 7415 | -0.112 | 0.0580 | No |
| 73 | ACTN3 | actinin alpha 3 [Source:HGNC Symbol;Acc:HGNC:165] | 7557 | -0.124 | 0.0520 | No |
| 74 | H4C7 | H4 clustered histone 7 [Source:HGNC Symbol;Acc:HGNC:4792] | 7607 | -0.127 | 0.0534 | No |
| 75 | C1R | complement C1r [Source:HGNC Symbol;Acc:HGNC:1246] | 7716 | -0.135 | 0.0505 | No |
| 76 | H2BC14 | H2B clustered histone 14 [Source:HGNC Symbol;Acc:HGNC:4750] | 8166 | -0.168 | 0.0223 | No |
| 77 | H3C1 | H3 clustered histone 1 [Source:HGNC Symbol;Acc:HGNC:4766] | 8270 | -0.177 | 0.0215 | No |
| 78 | ACTN1 | actinin alpha 1 [Source:HGNC Symbol;Acc:HGNC:163] | 8385 | -0.184 | 0.0201 | No |
| 79 | IL10 | interleukin 10 [Source:HGNC Symbol;Acc:HGNC:5962] | 8855 | -0.221 | -0.0074 | No |
| 80 | C8G | complement C8 gamma chain [Source:HGNC Symbol;Acc:HGNC:1354] | 8871 | -0.223 | 0.0005 | No |
| 81 | H2BC8 | H2B clustered histone 8 [Source:HGNC Symbol;Acc:HGNC:4746] | 8914 | -0.225 | 0.0064 | No |
| 82 | H2BC4 | H2B clustered histone 4 [Source:HGNC Symbol;Acc:HGNC:4757] | 8948 | -0.227 | 0.0132 | No |
| 83 | RO60 | "Ro60, Y RNA binding protein [Source:HGNC Symbol;Acc:HGNC:11313]" | 8968 | -0.230 | 0.0211 | No |
| 84 | H3C2 | H3 clustered histone 2 [Source:HGNC Symbol;Acc:HGNC:4776] | 8970 | -0.230 | 0.0304 | No |
| 85 | H2AX | H2A.X variant histone [Source:HGNC Symbol;Acc:HGNC:4739] | 8979 | -0.230 | 0.0392 | No |
| 86 | SNRPB | small nuclear ribonucleoprotein polypeptides B and B1 [Source:HGNC Symbol;Acc:HGNC:11153] | 9085 | -0.239 | 0.0408 | No |
| 87 | FCGR3B | Fc fragment of IgG receptor IIIb [Source:HGNC Symbol;Acc:HGNC:3620] | 9341 | -0.259 | 0.0314 | No |
| 88 | HLA-DPA1 | "major histocompatibility complex, class II, DP alpha 1 [Source:HGNC Symbol;Acc:HGNC:4938]" | 9481 | -0.273 | 0.0317 | No |
| 89 | H3-3A | H3.3 histone A [Source:HGNC Symbol;Acc:HGNC:4764] | 9503 | -0.274 | 0.0413 | No |
| 90 | H2AC18 | H2A clustered histone 18 [Source:HGNC Symbol;Acc:HGNC:4736] | 9514 | -0.275 | 0.0518 | No |
| 91 | SNRPD1 | small nuclear ribonucleoprotein D1 polypeptide [Source:HGNC Symbol;Acc:HGNC:11158] | 9579 | -0.281 | 0.0583 | No |
| 92 | H3C11 | H3 clustered histone 11 [Source:HGNC Symbol;Acc:HGNC:4771] | 9808 | -0.302 | 0.0528 | No |
| 93 | C1S | complement C1s [Source:HGNC Symbol;Acc:HGNC:1247] | 10012 | -0.323 | 0.0502 | No |
| 94 | H3C4 | H3 clustered histone 4 [Source:HGNC Symbol;Acc:HGNC:4767] | 10258 | -0.347 | 0.0452 | No |
| 95 | ACTN4 | actinin alpha 4 [Source:HGNC Symbol;Acc:HGNC:166] | 10472 | -0.370 | 0.0438 | No |
| 96 | H2AC13 | H2A clustered histone 13 [Source:HGNC Symbol;Acc:HGNC:4725] | 11296 | -0.470 | -0.0013 | No |
| 97 | H2AC7 | H2A clustered histone 7 [Source:HGNC Symbol;Acc:HGNC:4729] | 11343 | -0.477 | 0.0147 | No |
| 98 | GRIN2B | glutamate ionotropic receptor NMDA type subunit 2B [Source:HGNC Symbol;Acc:HGNC:4586] | 11487 | -0.500 | 0.0240 | No |
| 99 | SSB | small RNA binding exonuclease protection factor La [Source:HGNC Symbol;Acc:HGNC:11316] | 11955 | -0.584 | 0.0114 | No |
| 100 | H4C2 | H4 clustered histone 2 [Source:HGNC Symbol;Acc:HGNC:4789] | 12400 | -0.707 | 0.0056 | No |
| 101 | HLA-DRB4 | "major histocompatibility complex, class II, DR beta 4 [Source:HGNC Symbol;Acc:HGNC:4952]" | 12636 | -0.815 | 0.0206 | No |
Table: GSEA details [plain text format]

  

Fig 2: KEGG\_SYSTEMIC\_LUPUS\_ERYTHEMATOSUS      
 Blue-Pink O' Gram in the Space of the Analyzed GeneSet

  

Fig 3: KEGG\_SYSTEMIC\_LUPUS\_ERYTHEMATOSUS: Random ES distribution      
 Gene set null distribution of ES for **KEGG\_SYSTEMIC\_LUPUS\_ERYTHEMATOSUS**
